# Geometric quantum discord of Heisenberg model with dissipative terms

**DOI:** 10.1038/s41598-020-67698-9

**Published:** 2020-07-02

**Authors:** Jin Liang, Chengwei Zhang

**Affiliations:** School of Mathematical Sciences, Shanghai Jiao Tong University, Shanghai, 200240 China

**Keywords:** Quantum information, Quantum metrology

## Abstract

In this paper, we study a system with two interacting qubits described by the Heisenberg model with dissipative terms, and analyze decay dynamics and the steady-state of geometric quantum discords. Our results indicate that we can ignore the interaction force in the *z*-direction and adjust the parameters to change the loss of quantum correlation with time when the initial state satisfies some conditions. Moreover, we show that after a long enough period of time, unlike other parameters, the energy and the intensity of the non-uniform magnetic field do not affect the steady-state.

## Introduction

Quantum theory has received extensive attention in recent years due to its wide usage in a large number of new technologies^1–4^. Quantum correlation, as one of the basic questions in quantum theory^5^, has a great development in recent years^6–8^. Quantum correlation has multiple levels, such as nonlocal^9^, steerable^10^ or entangled^11^, and a framework for measuring the quantum system has been built. After quantum discord was firstly proposed by Ollivier and Zurek^2^ and by Henderson and Vedral^12^ respectively, a series of discord-like quantum correlations were proposed which exhibit different properties when characterizing the dynamics of the same quantum system and have become important resources in quantum theory. As one of discord-like quantum correlations, the geometric quantum discord, which will be used in this paper, has been widely concerned because it is easier to calculate and useful.

To gain a deeper understanding of the similarities and differences between such discord-like quantum correlations, we will use a model which can be solved exactly—the Heisenberg model in contact with a thermal reservoir. When describing the physical changes of a system, we often use the method of the master equation. And when the open system is weakly coupled with the environment, we can use the Markov approximation to get the approximation equation. It is known that the Heisenberg model is a classic model of the open quantum system. For example, it accurately explains the magnetic order in the ferromagnet^13^. So it has a high research value. Next, we will briefly explain the use of the Ket–Bra entangled state method to solve the time evolution of the equation and investigate its final steady-state properties. We will also study the changes in the coupling parameters that lead to changes in quantum correlation and the characterization of various geometric quantum discords.

## Geometric measure of quantum discord

Discussing the non-classicality of a composite system, which we collectively refer to as quantum correlation, is important to find a way to measure its deviation from the classical system. Considering the simplest case, for a system consisting of 2 bits, if part A is classic, we can say that it must be “on” or “off” (corresponding to state $| 1 \rangle _A,(| 0 \rangle _A )$. If the A part is in the state $| 1 \rangle _A$ $(or\,| 0 \rangle _A)$, then the density matrix of the whole system is $| 1 \rangle \langle 1 |_A \otimes \rho _B^1$ (or $| 0 \rangle \langle 0 |_A \otimes \rho _B^0$). Since the whole system may be in the quantum superposition state, the composite state $\rho _{AB}$ is, in general, a linear combination of the states above, that is,

$$\begin{aligned} \rho _{AB} =\sum _i p_i| i \rangle \langle i |_A\otimes \rho _{B} ^{i}, \end{aligned}$$

where $p_i$ is the corresponding probability, satisfying $\sum _i p_i=1$.

When the state $\rho$ can not be written in the above form, we know that it is not a classical-quantum state. The bigger the geometric distance of the state $\rho$ from the set of states above (i.e., the set of the classical-quantum states), the greater the quantum correlation. We say geometric quantum discord if it is defined in this way.

Since^14^, many geometric quantum discords have been used to describe the quantum correlation of quantum systems have been proposed. The following four geometric quantum discords are basic and important examples^5, 15, 16^:

### Hilbert–Schmidt distance discord

Hilbert–Schmidt distance discord is one of the metrics proposed to substitute quantum discord and is widely used because of its easy calculation. It has the following form

1 $$\begin{aligned} D_{Hs}\left( \rho \right) =\underset{\chi \in CQ}{min}d_{Hs}\left( \rho ,\chi \right), d_{Hs}\left( \rho ,\chi \right) =\left\| \rho -\chi \right\| _{2}^2, \end{aligned}$$

For the case of a two-level two-qubit, the density matrix $\rho$ has the following form

2 $$\begin{aligned} \rho =\frac{1}{4}\left(I\otimes I+X\sigma ^1 \otimes I+I\otimes Y\sigma ^2+\sum _{ij}t_{ij} \sigma _i^1 \otimes \sigma _j^2\right), \end{aligned}$$

where $$X=(x_1,x_2\cdot x_3), Y=(y_1,y_2,y_3), T=\{t_{ij}\} \ \ \sigma ^k=\begin{pmatrix} \sigma _x^k \\ \sigma _y^k \\ \sigma _z^k \end{pmatrix}$$.$\sigma _i^k,i\in \{x,y,z\}$ is the Pauli matrix of kth qubit. According to (1) and (2), we have

$$\begin{aligned} D_{Hs}\left( \rho \right) =\frac{1}{4} ( \left\| X \right\| _{2}^2+\left\| T \right\| _{2}^2+k_{max}), \end{aligned}$$

where $k_{max}$ is the maximum eigenvalue of the matrix $K=XX^{T}+TT^{T}$.

However, based on subsequent researches, we see that there is a problem about the Hilbert–Schmidt distance discord. From^17^, we know that

3 $$\begin{aligned} D_{Hs}\left( \rho _{AB} \otimes \rho _C \right) =D_{Hs}\left( \rho _{AB} \right) Tr \left( \rho _C^2 \right) , \end{aligned}$$

where $\rho _C$ is the density matrix of the part *C* (an auxiliary system). (3) shows that by adding or removing an auxiliary system (part *C*) that is not quantum related to the system, we may decrease or increase the Hilbert–Schmidt distance discord. However, physically speaking, the addition and removal of an ancilla is a reversible quantum operation on the unmeasured subsystem *B*, for which we require the quantum correlation never increase. The following three discords have no such a problem.

### Trace distance discord

According to the previous Hilbert–Schmidt distance discord, we examine its form

$$\begin{aligned} D_{n}\left( \rho \right) =\underset{\chi \in CQ}{min}d_{n}\left( \rho ,\chi \right) ,\ d_{n}\left( \rho ,\chi \right) =\left\| \rho -\chi \right\| _{n}^n. \end{aligned}$$

For the Schatten *n*-norm, it is known that the problem, about the Hilbert–Schmidt distance discord above, does not exist if and only if $n=1$ (we also call the 1-norm as trace norm^5^). That is trace distance discord, marked as $D_{Tr}\left( \rho \right)$. In^18^, it gives the analytical solution of the two-qubit.

### Hellinger distance discord

Hellinger distance discord has the following form

$$\begin{aligned} D_{He}\left( \rho \right) =\underset{\chi \in CQ}{min}d_{He}\left( \rho ,\chi \right) ,\ d_{He}\left( \rho ,\chi \right) =2\left[ 1-Tr(\sqrt{\rho }\sqrt{\chi })\right] . \end{aligned}$$

It also does not have the problem about the Hilbert–Schmidt distance discord above:

$$\begin{aligned} D_{He}\left( \rho _{AB} \otimes \rho _C \right) =D_{Hs}\left( \rho _{AB} \right) Tr \left( \sqrt{\rho _C}^2 \right) =D_{He}\left( \rho _{AB} \right) Tr \left( \rho _C \right) =D_{He}\left( \rho _{AB} \right) . \end{aligned}$$

### Bures distance discord

Bures distance discord has the following form

$$\begin{aligned} D_{Bu}\left( \rho \right) =\sqrt{(2+\sqrt{2})/2}\underset{\chi \in CQ}{min}d_{Bu}\left( \rho ,\chi \right) ,\ d_{Bu}\left( \rho ,\chi \right) =\sqrt{2} \left[ 1-Tr(\sqrt{\sqrt{\chi }\rho \sqrt{\chi }})\right] . \end{aligned}$$

According to^19^, it also avoids the problem about the Hilbert–Schmidt distance discord above. The closed formulae for Bures distance discords of 2-qubit Bell diagonal state are also given in^19, 20^.

## Heisenberg model with dissipative term and its solution

The master equation of the model^21^ takes the following form

4 $$\begin{aligned} \frac{d\rho }{dt}=-i[H,\rho ]+\sum _{n=1,2}{\mathscr{L}}_n\rho . \end{aligned}$$

The Hamilton in (4) is

$$\begin{aligned} H=B_1\sigma _{1}^{z}+B_2\sigma _{2}^{z}+J_{x} \sigma _{1}^{x} \sigma _{2}^{x}+J_{y} \sigma _{1}^{y} \sigma _{2}^{y}+J_{z} \sigma _{1}^{z} \sigma _{2}^{z}. \end{aligned}$$

$B_n\ (n=1,\ 2)$ is the transverse magnetic field on the *n*th site, and $J_a$ $(a\in \{x,y,z\}$) is the interaction of two spins. Set

$$\begin{aligned} J=J_x+J_y,\quad \Delta =J_x-J_y\ (-1\le \Delta \le 1),\quad B_+=(B_1+B_2)/2,\quad B_-=(B_1-B_2)/2, \end{aligned}$$

we call $\Delta$ as the anisotropy in the XY plane, $B_+$ as the uniform magnetic field intensity, and $B_-$ as the non-uniform magnetic field intensity.

The part of the dissipative term in (4) described by the Lindblad operator is

$$\begin{aligned} {\mathscr{L}}_{n} \rho =\sum _{i=1,2} \frac{\gamma _{n}}{2}\left( 2 c_{i, n} \rho c_{i, n}^{\dagger }-c_{i, n}^{\dagger } c_{i, n} \rho -\rho c_{i, n}^{\dagger } c_{i, n}\right) , \end{aligned}$$

where $c_{1, n}=(\overline{n}+1)^{1 / 2} \sigma _{n}^{-}, c_{2,n}=\overline{n}^{1 / 2} \sigma _{n}^{+}$, $\sigma _{n}^{+}/ \sigma _{n}^{-}$ is the lowering/raising operator on the *n*th site, $\overline{n}$ and $\gamma _{n}$ are the average thermal photons in the reservoir and the damping rates respectively. In this paper, let $\gamma _{1}=\gamma _{2}=\gamma$.

We will use the Ket–Bra entangled state method to study the above open quantum system. Set

5 $$\begin{aligned} | \eta \rangle =\sum _{m, n=0,1} | m n \rangle \otimes | \tilde{m}\tilde{n} \rangle , \end{aligned}$$

where $\{ | m n \rangle \}$ is a complete set of standard orthogonal bases, $m,\ n=0,\ 1$. Then the density matrix $\rho$ is converted to $| \rho \rangle$

$$\begin{aligned} | \rho \rangle =\rho | \eta \rangle =\sum _{m^{'} n^{'} m n } \rho _{m^{'} n^{'} m n} | m^{'} n^{'} \rangle \langle m n \Vert \eta \rangle =\sum _{m^{'} n^{'} m n } \rho _{m^{'} n^{'} m n} | m^{'} n^{'}\tilde{m} \tilde{n} \rangle \end{aligned}$$

by (5). Furthermore, we can prove

$$\begin{aligned} \sigma _{1}^{+} | \eta \rangle =\sigma _{1}^{+}\sum _{m, n=0,1} | m n \tilde{m}\tilde{n} \rangle =\sigma _{1}^{+}\sum _{m=0, n=0,1} | m n \tilde{m}\tilde{n} \rangle ==\tilde{\sigma }_{1}^{-}\sum _{m=1, n=0,1} | m n \tilde{m}\tilde{n} \rangle =\tilde{\sigma }_{1}^{-} | \eta \rangle , \end{aligned}$$

and similarly

$$\begin{aligned} \sigma _{i}^{\pm } | \eta \rangle =\tilde{\sigma }_{i}^{\mp } | \eta \rangle . \end{aligned}$$

Using the above relationship, the Ket–Bra entangled state is applied to both ends of the Eq. (4), and then we get

6 $$\begin{aligned} \frac{d}{d t}|\rho \rangle = {\mathscr{F}}\,|\rho \rangle \end{aligned}$$

Operator ${{\mathscr{F}}}$ in (6) can be written as

$$\begin{aligned} {\mathscr{F}} \equiv i\left( \tilde{H}-H\right) +\sum _{i=1,2}\left[ \beta \left( 2 \sigma _{i}^{+} \tilde{\sigma }_{i}^{+}-\sigma _{i}^{-} \sigma _{i}^{+}-\tilde{\sigma }_{i}^{-} \tilde{\sigma }_{i}^{+}\right) +\alpha \left( 2 \sigma _{i}^{-} \tilde{\sigma }_{i}^{-}-\sigma _{i}^{+} \sigma _{i}^{-}-\tilde{\sigma }_{i}^{+} \tilde{\sigma }_{i}^{-}\right) \right] . \end{aligned}$$

Because ${\mathscr{F}}$ is independent of time, we obtain the solution of the Eq. (6) as

$$\begin{aligned} | \rho _{t} \rangle =e^{{\mathscr{I}} t} | \rho _{0} \rangle , \end{aligned}$$

where $\alpha =\gamma ( \overline{n}+1 )$, and $\beta =\gamma \overline{n}$. According to the observation of ${\mathscr{F}}$, we see that the X part and the non-X part of the matrix evolve independently, that is, when the initial state is the X state, the result is also the X state. Now we only consider the X state. Let $| 00 \tilde{0}\tilde{0} \rangle ,| 01 \tilde{0}\tilde{1} \rangle ,| 10 \tilde{1}\tilde{0} \rangle ,| 11 \tilde{1}\tilde{1} \rangle ,| 00 \tilde{1}\tilde{1} \rangle ,| 01 \tilde{1}\tilde{0} \rangle$, $| 10 \tilde{0}\tilde{1} \rangle$, $| 11 \tilde{0}\tilde{0} \rangle$ be the basis. Then the matrix form of ${\mathscr{F}}$ is

$$\begin{aligned} \begin{pmatrix} {\begin{matrix} -\,4\beta &{}\quad 2\alpha &{}\quad 2\alpha &{}\quad 0 &{}\quad iJ\Delta &{}\quad 0 &{}\quad 0 &{}\quad -\,iJ\Delta \\ 2\beta &{}\quad -\,2\alpha -\,2\beta &{}\quad 0 &{}\quad 2\alpha &{}\quad 0 &{}\quad iJ &{}\quad -\,iJ &{}\quad 0\\ 2\beta &{}\quad 0 &{}\quad -\,2\alpha -\,2\beta &{}\quad 2\alpha &{}\quad 0 &{}\quad -\,iJ &{}\quad iJ &{}\quad 0\\ 0 &{}\quad 2\beta &{}\quad 2\beta &{}\quad -\,4\alpha &{}\quad -\,iJ\Delta &{}\quad 0 &{}\quad 0 &{}\quad iJ\Delta \\ iJ\Delta &{}\quad 0&{}\quad 0 &{}\quad -\,iJ\Delta &{}\quad -\,2\alpha -\,2\beta -\,2iB_+ &{}\quad 0 &{}\quad 0&{}\quad 0 \\ 0 &{}\quad iJ &{}\quad -\,iJ &{}\quad 0 &{}\quad 0 &{}\quad -\,2\alpha -\,2\beta -\,2iB_-\, &{}\quad 0 &{}\quad 0 \\ 0 &{}\quad -\,iJ &{}\quad iJ &{}\quad 0 &{}\quad 0 &{}\quad 0 &{}\quad -\,2\alpha -\,2\beta +2iB_-\, &{}\quad 0 \\ -\,iJ\Delta &{}\quad 0 &{}\quad 0 &{}\quad iJ\Delta &{}\quad 0 &{}\quad 0 &{}\quad 0 &{}\quad -\,2\alpha -\,2\beta +2iB_+ \end{matrix}} \end{pmatrix}. \end{aligned}$$

Thus, we see that the value of $J_z$ has no effect on the time evolution of the equation. In fact, $J_z$ only affects the non-X part. In other words, the model is simplified to the Heisenberg XY model. Finally, change $| \rho (t) \rangle$ back to $\rho (t)$. Then, we get the solution of the equation.

## Dynamics of geometric quantum discord

We study the quantum correlation of the model. We will use the magnitude of the quantum correlation as a signal strength. Consider the initial state $|\varphi ^+ \rangle =|00\rangle +|11\rangle$ and $|\psi ^+ \rangle =|01\rangle +|10\rangle$. Then, $|\varphi ^- \rangle =|00\rangle -|11\rangle$ and $|\psi ^- \rangle =|01\rangle -|10\rangle$ as the initial state are like $|\varphi ^+ \rangle$ and $|\psi ^+ \rangle$, respectively. When $\Delta =J=0$, the form of $e^{{\mathscr{F}}t}$ is now reduced to

$$\begin{aligned} \begin{pmatrix} *&{}\quad *&{}\quad *&{}\quad *&{}\quad 0 &{}\quad 0 &{}\quad 0 &{}\quad 0\\ *&{}\quad *&{}\quad *&{}\quad *&{}\quad 0 &{}\quad 0 &{}\quad 0 &{}\quad 0\\ *&{}\quad *&{}\quad *&{}\quad *&{}\quad 0 &{}\quad 0&{}\quad 0 &{}\quad 0\\ *&{}\quad *&{}\quad *&{}\quad *&{}\quad 0 &{}\quad 0&{}\quad 0 &{}\quad 0\\ 0&{}\quad 0 &{}\quad 0 &{}\quad 0 &{}\quad e^{-2(a+b+iB_+)t} &{}\quad 0 &{}\quad 0 &{}\quad 0\\ 0&{}\quad 0 &{}\quad 0 &{}\quad 0 &{}\quad 0 &{}\quad e^{-2(a+b+iB_-)t} &{}\quad 0 &{}\quad 0\\ 0 &{}\quad 0 &{}\quad 0 &{}\quad 0 &{}\quad 0 &{}\quad 0 &{}\quad e^{-2(a+b-iB_-)t} &{}\quad 0\\ 0&{}\quad 0 &{}\quad 0 &{}\quad 0 &{}\quad 0 &{}\quad 0 &{}\quad 0 &{}\quad e^{-2(a+b-iB_+)t} \end{pmatrix}. \end{aligned}$$

Where $*$ represents a variable that depends only on $\alpha , \beta$, and *t*. Corresponding $\rho$ is

$$\begin{aligned} \begin{pmatrix} \rho ^{\{\alpha ,\beta ,t\}}_{11} &{}\quad 0 &{}\quad 0&{}\quad e^{-2(a+b+iB_+)t}\rho _{14}\\ 0 &{}\quad \rho ^{\{\alpha ,\beta ,t\}}_{22}&{}\quad e^{-2(a+b+iB_-)t}\rho _{23} &{}\quad 0\\ 0 &{}\quad e^{-2(a+b-iB_-)t}\rho _{32}&{}\quad \rho ^{\{\alpha ,\beta ,t\}}_{33} &{}\quad 0\\ e^{-2(a+b-iB_+)t} \rho _{41} &{}\quad 0 &{}\quad 0&{}\quad \rho ^{\{\alpha ,\beta ,t\}}_{44} \end{pmatrix}. \end{aligned}$$

One property of various quantum discords is invariance under at least one local unitary operation, i.e. $D(\rho _{AB})=D((U_A \otimes U_B)\rho _{AB}(U_A^\dagger \otimes U_B^\dagger ))$, where $U_n$ is the local unitary operation on subsystem *n*. If we take

$$\begin{aligned} U_A=\begin{pmatrix} 0 &{}\quad e^{iB_1/2}\\ e^{-iB_1/2} &{}\quad 0 \end{pmatrix},\ U_B=\begin{pmatrix} 0 &{}\quad e^{iB_2/2}\\ e^{-iB_2/2} &{}\quad 0 \end{pmatrix} \end{aligned}$$

then we deduce that

$$\begin{aligned} (U_A \otimes U_B)\rho _{AB}(U_A^\dagger \otimes U_B^\dagger )= \begin{pmatrix} \rho ^{\{\alpha ,\beta ,t\}}_{11} &{}\quad 0 &{}\quad 0&{}\quad e^{-2(a+b)t}\rho _{14}\\ 0 &{}\quad \rho ^{\{\alpha ,\beta ,t\}}_{22}&{}\quad e^{-2(a+b)t}\rho _{23} &{}\quad 0\\ 0 &{}\quad e^{-2(a+b)t}\rho _{32}&{}\quad \rho ^{\{\alpha ,\beta ,t\}}_{33} &{}\quad 0\\ e^{-2(a+b)t} \rho _{41} &{}\quad 0 &{}\quad 0&{}\quad \rho ^{\{\alpha ,\beta ,t\}}_{44} \end{pmatrix}. \end{aligned}$$

Therefore, the value of the geometric quantum discord is only related to $\alpha , \beta$ (both related to $\overline{n}$) and *t*.

When $\Delta =0$, the model is reduced to the Ising model. Let us observe the relationship between the average photon number and the magnetic field strength of the environment. Similar to the previous method, we see that $B_1$ can still be eliminated by the local unitary transform. But $B_2$ has an effect on the model.

From^5, Section 3.3.5^, we know that an interesting link to the Hilbert–Schmidt based geometric measure was also established

$$\begin{aligned} D_{He}\left( \rho _{A B}\right) =2-2 \sqrt{1-D_{Hs}(\sqrt{\rho _{A B}})} \end{aligned}$$

and^20^

$$\begin{aligned} D_{bures}(\rho )=\frac{1}{2}(1-{\text {tr}} \Lambda )+\sum _{l=1}^{n_{B}} \lambda _{l} \end{aligned}$$

where $\lambda _{1} \ge \cdots \ge \lambda _{n_{B}}$ and $n_{B}$ is the dimension of $\Lambda$

$$\begin{aligned} \Lambda ({\vec{u}})=\sqrt{\rho } \sigma _{{\vec{u}}} \otimes 1 \sqrt{\rho } \end{aligned}$$

with ${\vec{u}} \in {\mathbb {R}}^{3}$ and $\Vert {\vec{u}}\Vert =1$. Based on these facts, we get the idea to draw the following four figures by some numerical method.

**Figure 1:**
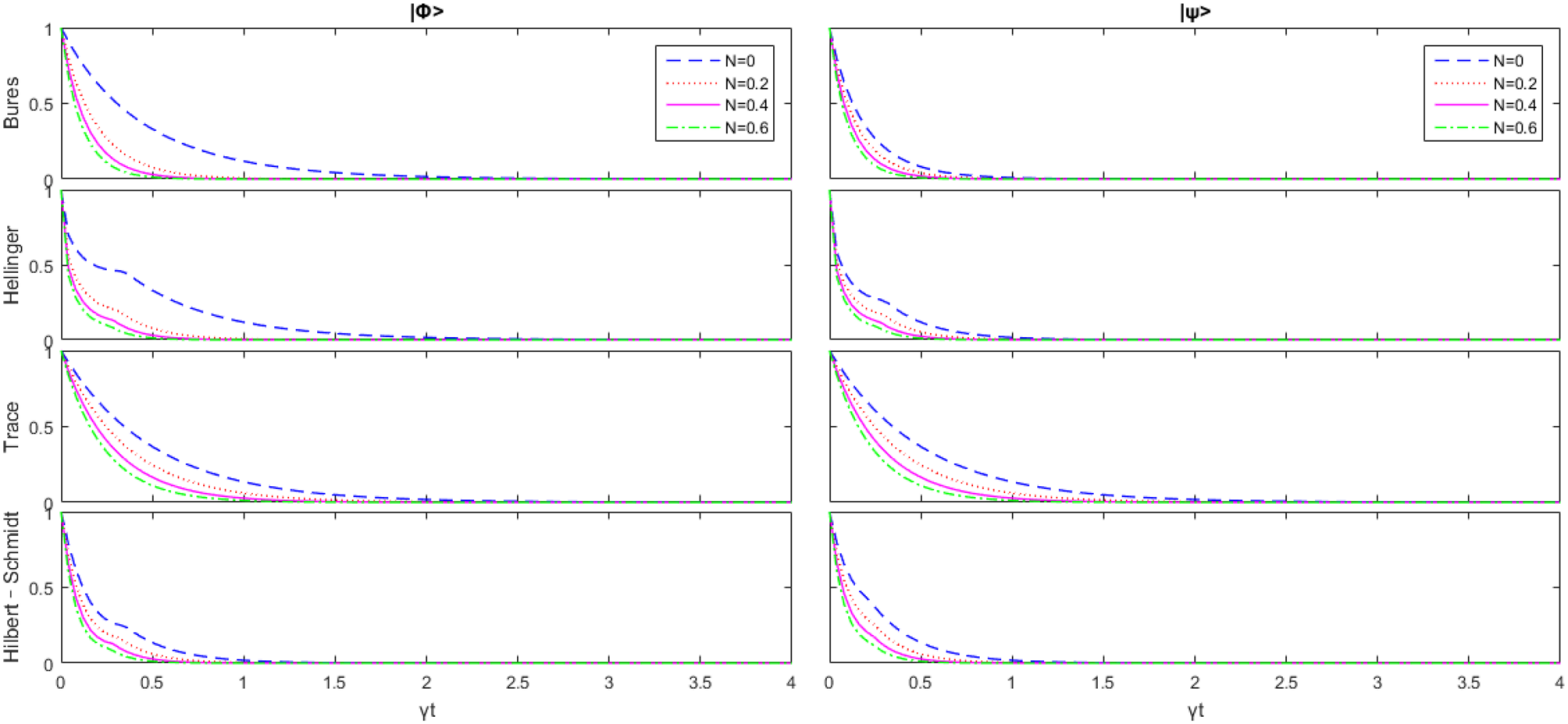
Change of the initial state $|\psi \rangle$ and $|\varphi \rangle$ with respect to $\overline{n}$ as $B_+=B_-=\Delta =0$, and *J* is the unit.

From Fig. 1, we can see that with the increase of $\overline{n}$, all kinds of geometric quantum discords are reduced, and that there are some obvious differences in these four geometric quantum discords. Unlike trace distance discord and the Bures distance discord, there is a significant sharp transition between the Hellinger distance discord and the Hilbert–Schmidt distance discord. The rate of decline before and after the change point is abrupt. This phenomenon happens in both initial states. It means that the Hellinger distance discord and the Hilbert–Schmidt distance discord keep clearer signals in short time but rapid decay after abrupt change point.

**Figure 2:**
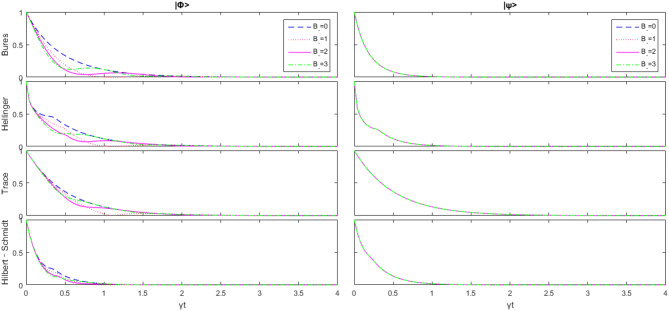
Change of the initial state $|\psi \rangle$ and $|\varphi \rangle$ with respect to $B_-$ as $B_+=\overline{n}=\Delta =0$, and *J* is the unit.

As can be seen from Fig. 2, the selection of initial values has a significant impact on the evolution of time. $$|\psi \rangle $$ does not change with non-uniform magnetic field strength $$B_-$$, while $$|\varphi \rangle $$ is significantly different. For $$|\varphi \rangle $$, at the beginning, it is not affected by $$B_-$$, but something different occurs as time goes on. Actually, we find that the geometric quantum discord intensity does not decrease monotonically with time. Furthermore, when considering a pair of qubits exposed to local noisy environments, zero quantum correlation can occur in a finite time, differently from the usual local decoherence in asymptotic time. The occurrence of this phenomenon is named sudden death. We notice that sudden death and sudden rebirth occur when $$ B_- =1$$. This implies that the quantum correlation completely disappears after a short period of evolution and appears again after sudden death. This phenomenon does not occur in other values of $$B_-$$. Finally, if it goes long enough, all the geometric quantum discords still converge to the same point (zero). In this case, regardless of the magnetic field intensity, the model will eventually be completely decoherent.

When $\Delta =1$, the model is reduced to the Heisenberg XX model. Next, we shall observe its relationship with the magnetic field intensity.

**Figure 3:**
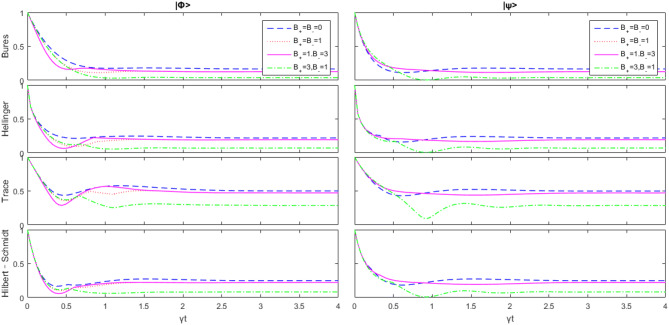
Change of the initial state $|\psi \rangle$ and $|\varphi \rangle$ with respect to $B_-$ as $B_+=\overline{n}=0 ,\Delta =1$, and *J* is the unit.

From Fig. 3, we can see that after a long enough period of time, the first time value does not tend to zero. This means that the interaction between subsystems can effectively prevent the decoherence process. And for the same parameters but different initial states, the same geometric quantum discord tends to the same value. When time changes, $|\psi \rangle$ is more sensitive to $B_+$ , and $|\varphi \rangle$ is more sensitive to $B_-$. In various geometric quantum discords, the effect of the trace distance discord on strength maintenance is the best. When $B_+=3,\ B_-=1$, and $|\psi \rangle$ is the initial value, there are almost sudden deaths in other geometric quantum discords (all have $D(\rho )<0.01$), but for the trace distance discord $D_{Tr}(\rho )>0.05$. This also suggests that the trace distance discord is more resistant to external perturbations.

**Figure 4:**
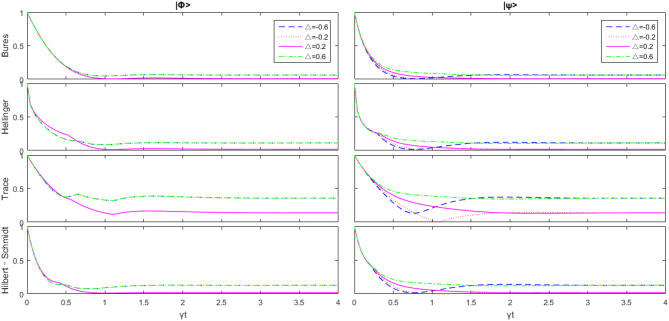
Change of the initial state $|\psi \rangle$ and $|\varphi \rangle$ with respect to $\Delta$ as $B_-=B_+=1,\overline{n}=0$, and *J* is the unit.

Now for the Heisenberg XY model, we mainly consider the change of $\Delta$. From Fig. 4, we can see that the effect is similar to $B_-$. It is not significant at first, and then has different characteristics according to the initial state: $|\varphi \rangle$ only changes according to $|\Delta |$, and $| \psi \rangle$ is different, even when the trace distance discord has sudden death. But eventually $|\psi \rangle$ is the same as $|\varphi \rangle$ and is only affected by $|\Delta |$ instead of $\Delta$.

By the discussion and figures above and known relevant information, we see that each of the four types of discord measures above has its own characteristics when dealing with different models, but the trace distance discord has the least loss of signal strength compared to others in most situations especially for the models in this paper, that is, the trace distance discord is more robust. The magnetic field intensity has great potential to control the associated decay speed and attenuation form. In next section, we will analyze the steady-state with the trace distance discord, and we will show the impact of $B_+$ and $B_-$ to the steady-state.

## Steady-state

Consider the form of $e^{{\mathscr{F}}t}$ when $t \rightarrow \infty$ and the initial state is the X state. The eigenvalues of ${\mathscr{F}}$ are $0,-4\iota , -2\iota , -2\iota , -2 (\iota \pm i\kappa _1), -2 (\iota \pm i \kappa _2)$, where $\kappa _1=\sqrt{B_2^2 + J^2},\kappa _2=\sqrt{B_1^2 + (J\Delta )^2},\iota =\alpha +\beta$. All the real part of the eigenvalues are no more than 0. When $t \rightarrow \infty$ and the real part of $\lambda _i$ is less than 0, we can assert $|e^{\lambda _i t}|=0$. So we only need to consider the case of $\lambda =0$, then the form of $e^{{\mathscr{F}}t}$ is

$$\begin{aligned} \begin{pmatrix} \rho _1 &{}\quad \rho _1 &{}\quad \rho _1 &{}\quad \rho _1 &{}\quad 0 &{}\quad 0 &{}\quad 0 &{}\quad 0\\ \rho _2 &{}\quad \rho _2 &{}\quad \rho _2 &{}\quad \rho _2 &{}\quad 0 &{}\quad 0 &{}\quad 0 &{}\quad 0\\ \rho _3 &{}\quad \rho _3 &{}\quad \rho _3 &{}\quad \rho _3 &{}\quad 0 &{}\quad 0 &{}\quad 0 &{}\quad 0\\ \rho _4 &{}\quad \rho _4 &{}\quad \rho _4 &{}\quad \rho _4 &{}\quad 0 &{}\quad 0 &{}\quad 0 &{}\quad 0\\ \rho _5 &{}\quad \rho _5 &{}\quad \rho _5 &{}\quad \rho _5 &{}\quad 0 &{}\quad 0 &{}\quad 0 &{}\quad 0\\ 0 &{}\quad 0 &{}\quad 0 &{}\quad 0 &{}\quad 0 &{}\quad 0 &{}\quad 0 &{}\quad 0 \\ 0 &{}\quad 0 &{}\quad 0 &{}\quad 0 &{}\quad 0 &{}\quad 0 &{}\quad 0 &{}\quad 0 \\ \rho _6 &{}\quad \rho _6 &{}\quad \rho _6 &{}\quad \rho _6 &{}\quad 0 &{}\quad 0 &{}\quad 0 &{}\quad 0 \end{pmatrix}, \end{aligned}$$

where

$$\begin{aligned} \rho _1= & {} \frac{4\alpha ^2(\iota ^2+B_+^2)+(\iota \Delta J)^2}{4\iota ^2\zeta }, \quad \rho _2=\frac{4\alpha \beta (\iota ^2+B_+^2)+(\iota \Delta J)^2}{4\iota ^2\zeta },\\ \rho _3= & {} \frac{4\alpha \beta (\iota ^2+B_+^2)+(\iota \Delta J)^2}{4\iota ^2\zeta }, \quad \rho _4=\frac{4\beta ^2(\iota ^2+B_+^2)+(\iota \Delta J)^2}{4\iota ^2\zeta },\\ \rho _5= & {} \frac{(\alpha -\beta )(i\iota +B_+)\Delta J}{2\iota \zeta }, \quad \rho _6=\frac{(\alpha -\beta )(-i\iota +B_+)\Delta J}{2\iota \zeta }=\rho _5^*, \end{aligned}$$

and $\zeta =\iota ^2+\kappa _2^2$. So for any initial state $\rho$, we have $tr(\rho )=1$, that is, $\sum _{i=1}^4|\rho ^i(0)\rangle =1,|\rho ^i(0)\rangle$ is the *i*th component of $|\rho (0)\rangle$. Therefore, the final state $\rho (\infty )$ should be

$$\begin{aligned} \begin{pmatrix} \rho _1 &{}\quad 0 &{}\quad 0 &{}\quad \rho _5 \\ 0 &{}\quad \rho _2 &{}\quad 0 &{}\quad 0\\ 0 &{}\quad 0 &{}\quad \rho _3 &{}\quad 0\\ \rho ^*_5 &{}\quad 0 &{}\quad 0 &{}\quad \rho _4 \end{pmatrix}. \end{aligned}$$

We can see that the form is independent of the size of $B_-$, *J* and the initial state, which is consistent with the conclusions of the previous section. Take the trace distance discord as an example and use the symbols in^18^. From the fact that

$$\begin{aligned} r_1= & {} r_2=2|\rho _{41}(\infty )|=\frac{(\alpha -\beta )\sqrt{(\iota ^2+B_+^2)}|\Delta |J}{\iota \zeta },\\ r_3= & {} \frac{(\alpha -\beta )^2(\iota ^2+B_+^2)}{\iota ^2\zeta },\quad \chi _{a3}=\frac{(\alpha ^2-\beta ^2)(\iota ^2+B_+^2)}{\iota ^2\zeta }, \end{aligned}$$

we know that there is always $\chi _{a3} \ge r_3$ and we can get $D_{Tr}(\infty )=|r_1|$, and that when $\overline{n}$ increases, the maximum value of $D_{Tr}(\infty )$ is reduced.

When $\iota ^2+B_+^2$ equals to $\Delta ^2J^2$, we get the maximum value $1/(4\overline{n}+2)$. Therefore, for any initial state $\rho$, we can see that when the value of the initial state of the trace distance discord is greater than this value, it must be reduced.

## Discussion

In this paper, we discuss several geometric quantum discords over time for the Heisenberg model with respective to thermal reservoirs for the initial state of the *X* state, and find that in such initial state, the *z*-direction interaction does not affect the result at all. Then we study the effects of $\overline{n}$, $B_+$, $B_-$, and $\Delta$ on time evolution, and observe that in some cases sudden death and rebirth occur. By comparison, we see that the trace distance mismatch has better robustness. Furthermore, we investigate their steady-state and show that the initial state *J* and $B_-$ do not affect the results also. Finally, the problem when trace distance discord can achieve its maximum is discussed as well.

Compared with previous studies, we consider the model with non-uniform magnetic field which broadens largely the scope of application of the model. Through our analysis, we find that in the XYZ model, if the initial state is the X state, it can be reduced to the XY model. With the change over time of geometric quantum discords under different parameters, new characteristics are revealed.
